# Vitamin D deficiency in rheumatoid arthritis patients of India – a single-arm meta-analysis

**DOI:** 10.4314/ahs.v23i1.84

**Published:** 2023-03

**Authors:** Debdipta Bose, Renju Ravi, Miteshkumar Maurya, R Legha, Mahanjit Konwar

**Affiliations:** 1 Department of Clinical Pharmacology, Seth GS Medical college & KEM Hospital, Mumbai, India; 2 Department of Clinical Pharmacology, Faculty of Medicine, Jazan University, Jazan 45142, Saudi Arabia; 3 Department of Medicine, Travancore Medical college, Kollam, Kerala, India

**Keywords:** Vitamin D level, disease activity score, Vitamin D supplementation, Vitamin D insufficiency

## Abstract

**Background:**

Vitamin D deficiency is commonly seen in patients with rheumatoid arthritis (RA).

**Objectives:**

This meta-analysis is aimed to determine the prevalence of Vitamin D deficiency in RA patients in India and also to evaluate the association between vitamin D level and disease activity.

**Methods:**

The relevant works of literature were identified through multiple databases and data was extracted from eligible studies independently. A single-arm meta-analysis was performed to estimate the prevalence of Vitamin D deficiency in RA patients in an Indian setup and its association with disease activity. A total of 15 studies was included in the analyses.

**Results:**

The mean serum vitamin D level was 19.99 ng/ml [95% CI 16.49-24.23]. The proportion of patients with low vitamin D level was 0.80 [95% CI 0.65- 0.90], Vitamin D deficiency was 0.56 [95% CI 0.31-0.77] and vitamin D insufficiency was 0.20 [95% CI 0.12- 0.32]. A negative relationship was seen with serum vitamin D and disease activity score.

**Conclusions:**

The results demonstrate significant low levels of serum vitamin D levels in patients with RA and established a negative correlation of Vitamin D with RA disease activity. The current evidence suggests a rationale for Vitamin D supplementation in the management of RA.

## Introduction

Rheumatoid arthritis [RA] is a chronic inflammatory connective tissue disease of unknown etiology with varied clinical presentations. Rheumatoid arthritis impacts the quality of life adversely in the majority of patients. The disease commonly affects females of the age group of 30-50 years.^1^

The etiopathogenesis of RA involves multiple mechanisms in the development of the disease. The 1,25-dihydroxy vitamin D3 or calcitriol, the biologically active metabolite of vitamin D, is considered one of the important environmental factors contributing to the genesis of many autoimmune diseases.^2^ Calcitriol inhibits pro-inflammatory Th1 and Th17 responses and promotes Th2 and T-reg responses, leading to regulation of the immune response of T effector cells^2^,^3^, mechanisms that are implicated in the pathogenesis of RA. Furthermore, Vitamin D also has a scavenging effect on the reactive oxygen species (ROS). Interaction of 1,25(OH)2D with Vitamin D receptor [VDR] is important for mitochondrial integrity and respiration. Moreover, the Vitamin D signalling pathway confers protection against DNA damage induced by the overproduction of reactive oxygen species and elevated mitochondrial respiration.^4^,^5^

Nonetheless, it also exerts immunomodulation via the nuclear vitamin D Receptor (VDR) expressed in antigen-presenting cells (APC) and activated T/B cells. These immunomodulatory activities of vitamin D might be particularly efficient in RA patients and support a therapeutic role of 1,25-dihydroxy vitamin D3 in such a disease, and it was found to be associated with a lower risk of RA with greater intake of Vitamin D.^3^

There are established clinical and biochemical measures such as Disease Activity Score-28 (DAS), rheumatoid factor (RF) and anti-citrullinated protein antibodies (ACPA) which are prognostic of the severity of the disease.^6^ 25-hydroxyvitamin D3 (25(OH)D) is the primary circulating form of vitamin D and is considered to be the best indicator of Vitamin D status in humans. However, serum (25(OH)D) level is yet to be established as a prognostic marker of Rheumatoid arthritis.^3^,^6^ A comprehensive literature search revealed that many studies identified a negative correlation between Vit D and disease activity in RA, on contrary, few studies refuted this relation.^7^ Meta-analysis of the studies with 25(OH)D levels and disease activity in RA found that Vitamin D deficiency is associated with higher disease activity in RA patients. 8

The prevalence of RA in India ranges from 0.28-0.7% whereas the prevalence of Vitamin D deficiency (<20ng/ml) is quite higher in the Indian population (50-94%). 9–11 The relationship between the levels of 25 hydroxyvitamin D (25(OH)D) and the severity of RA is a subject of immense interest and therapeutic implications. Many studies in India have evaluated the serum 25(OH)D levels in RA patients, however, there is no collective data generated from these studies. Hence, the present study was envisaged to conduct a single-arm meta-analysis to understand the prevalence of Vitamin D deficiency in Indian RA patients and to ascertain the association between 25(OH)D levels and disease activity.

## Methods

We performed a systematic review and single-arm meta-analysis to investigate the vitamin D deficiency in rheumatoid arthritis patients of India following an a priori study protocol registered with the International Prospective Register of Systematic Reviews - PROSPERO [Registration no: CRD - 42022297923]. The study was reported according to the Preferred Reporting Items for Systematic Reviews and Meta-Analyses (PRISMA) statement - 2020.^12^ [Supp.table-3]

### Search strategy and data extraction

We searched the PubMed, Cochrane, Embase and Google scholar databases with search filter for keywords such as “25(OH)D levels”, “25 Hydroxyvitamin D level”, “Vitamin D”, “India” “Prevalence”,” Incidence” and ‘Rheumatoid Arthritis”. The search was conducted till 30^th^ May 2021. All types of clinical studies such as randomized controlled trials [RCT], cohort studies and case-control studies were included. We restricted our search to English Language only. Data were extracted by two reviewers (MK and MM) and the following information was collected from each study, first author's, sample size, type of study, serum 25(OH)D and anti-cyclic citrullinated peptides [anti-CCP] levels and Disease Activity Score-28 item questionnaire^30^ score. Any discrepancies were resolved by consensus in consultation with a third reviewer (RR). Studies were considered ineligible if the above information is not present and the RA diagnosis criteria are not mentioned.

### Endpoints and quality assessments of studies

We have used the following outcome measures a) mean serum 25(OH)D levels in RA patients and proportion of RA patients with b) low 25(OH)D levels(<30ng/dl) which is further divided as c) Vitamin D deficiency(<20ng/dl) d) Vitamin D insufficiency (20-30ng/dl).^13^, ^14^

The quality of studies was assessed by using Cochrane Collaboration's tool for randomized studies and Newcastle-Ottawa Scale (NOS) for observational studies.^15^,^16^

### Statistics Analysis

Two authors [MK and DB] analysed the data. The single-arm meta-analyses were performed by using software 'R version 4.05’ with “metaprop” and “metamean” packages.^17^ The pooled mean [25(OH)D levels] and proportions [incidence of Vitamin D deficiency and insufficiency] with corresponding 95% Confidence Intervals (CI) were calculated by using both fixed and the random-effects model. The outcomes were presented graphically by forest plot. Statistical heterogeneity was estimated through the I^2^ statistics and judged to be either high (>75%), moderate (25%-75%) or low (<25%).^18^ Egger test was performed to evaluate for publication bias for the outcome with at least 10 studies. 19 The analysis of variance [ANOVA] was used to measure the association between DAS scores [ mild (<3.1), moderate (3.1 to 5.2) and severe (>5.2) and 25(OH)D levels. A two-tailed p-value less than 0.05 represented statistical significance.

## Results

**Suppl. Figure 1:**
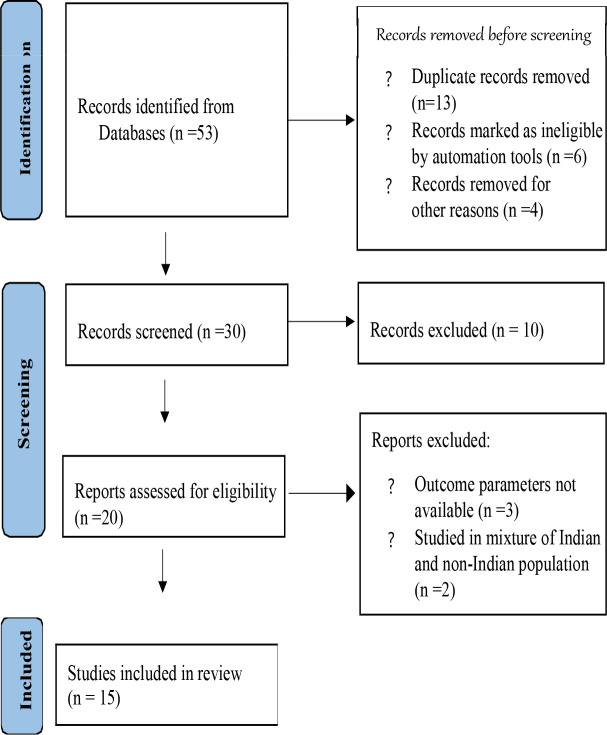
PRISMA flow diagram

Our literature search yielded 53 articles of which 20 articles underwent full-text review and finally 15 studies 20–34 were included in the meta-analysis. The process of inclusion is demonstrated in the Preferred Reporting Items for Systematic Reviews and Meta-Analyses [PRISMA] flow diagram [Suppl. Fig 1].

### Characteristics of the studies

#### Trial characteristics

Out of 15 studies, 8 [53.3%] were case-control studies, 4 [26.6%] were cross-sectional and three [20.1%] were randomized controlled trial. The sample size was less than 50 in 3 [20%] studies and the remaining 12 [80%] studies had a sample size of more than 50. [Table 1]

**Table 1:** Characteristics of included studies

Study ID	Study design	Sample size	Age [years]	Gender	Vit D level (ng/ml)
Agarwal *et* *al* 2020 20	Cross-sectional	30	39.9 ± 12.1	F – 27 M – 3	18.93 ± 6.64
Borukar *et* *al* 2017 21	Case-control	92	Case - 44.62 ± 13.47 Control - 38.55 ± 13.27	F- 80 M - 12	Case - 64.03 ± 41.36 Control - 58.90 ± 24.27
Bhimwal *et* *al* 2017 22	Case-control	50	NA	F - 28 M - 22	NA
Chandrashekhar *et* *al* 2015 (a) 23	Randomized	138	High DAS - 47.2 ± 12.2 Low DAS - 50.0 ± 11.5	F – 119 M - 19	High DAS - 10.5 ± 5.91 Low DAS - 13.0 ± 6.35
Gopinath K *et* *al* 2011 24	Randomized	121	Vit D - 44.86± 12.3 Ca-Carb- 44.90 ± 12.5	F – 91 M - 30	NA
Jagtap et al 2017 25	Case-control	26	Case - 47.33± 7.28 Control - 45.04±10.24	F - 15 M - 11	Case - 21.36±13.03 Control - 18.41±13.66
Latief *et al* 2017 [26]	Case-control	50	Case - 35.36 ± 3.18 Control - 34.52 ± 3.17	NA	Case - 18.41 ± 7.10 Control - 22.32 ± 4.80
Mateen *et al* 2017 27	Case-control	100	Case - 40.06±10.63 Control - 40.92±10.24	NA	Case - 12.66±4.81 - RA, Control - 37.88±9.78
Marangmei *et* *al* 2020 [28]	Cross- sectional	52	49.63±12.60	F - 47 M – 5	26.56±11.29
Meena *et al* 2018 29	Case-control	50	Case - 44.92 ± 13.06 Control - 44.02 ± 11.65	F – 43 M – 7	Case - 21.05 ± 10.02 Control - 32.87 ± 14.16
Mukherjee *et* *al* 2019 30	Randomized	150	Vit D + Ca- 36.7±10.9 Ca - 38.8±13.5	F – 125 M – 25	NA
Pallivalappil *et* *al* 2021 [31]	Cross sectional	60	42.5 ± 8.3	F - 48 M - 12	Case - 15.8 Control - 21.54
Rai *et al* 2017 32	Case-control	80	Case - 44.35 ± 10.05 Control - 36.35 ± 9.45	F - 68 M – 12	Case - 14.14 ± 10.26 Control - 12.31 ± 8.07
Ramu *et al* 201 33	Cross sectional	40	42.2 ± 12.3	F - 37 M - 3	30 (SD not mentioned)
Sharma *et al* 2014 34	Case-control	160	Case - 40.97 ± 12.52 Control - 42.63 ± 12.66	NA	Case - 17.20 + 10.74 Control - 22.39 +14.03

M – male, F – Female, NA – not available, SD – Standard deviation

#### Participant characteristics

Among the 15 studies, in 12 [80%] studies the mean age was more than 40 years and in three studies [20%] the mean age was more than 35 years. Female preponderance was noted in all 15 studies. The mean 25(OH)D level was less than 20 ng/ml for RA cases in the six [40%] studies with 480 patients and three [20%] studies of 170 patients had a mean Vitamin D level of more than 20 ng/ml for RA cases. [Table 1]

### Risk of bias assessment

**Suppl. Figure 2:**
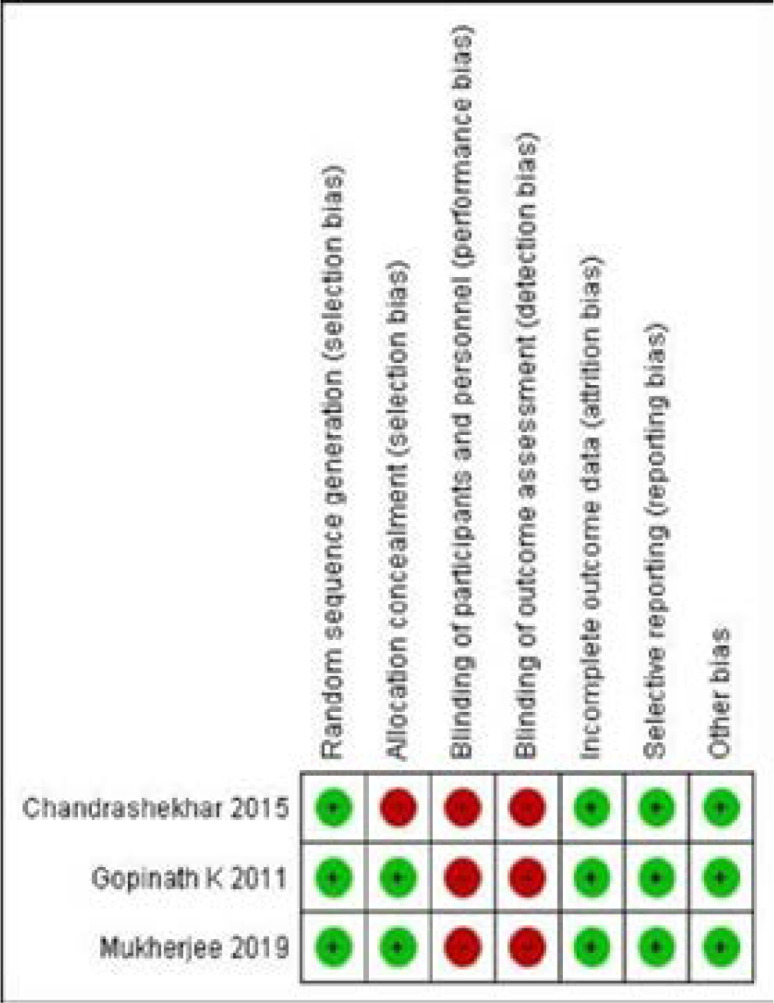
Risk of bias summary

The quality assessment of the studies was based on NOS and Cochrane collaboration tools. The results are depicted in Suppl. table 1, Suppl. table 2 and Suppl. fig. 2.

**Suppl. Table 1:** Quality assessment of Case control studies using NEWCASTLE OTTAWA SCALE (NOS)

Study ID [Author]	Selection				Comparability	Exposure			Overall Quality
	Is the Case Definition Adequate	Representativeness of the Cases	Selection of Controls	Definition of Controls	Comparability of cases and controls on the basis of the design or analysis	Ascertainment of exposure	Same method of ascertainme nt for cases and controls	Non- Response rate (dropouts)	
Borukar *et* *al* 2017	American College of Rheumatology (ACR) criteria and radiological analysis used for definition*	Recruitment of the patients was from OPD of Sir H. N. Reliance Foundation Hospital and Research Centre. *	The 50 controls were enrolled from the same hospital*	The 50 controls were enrolled*	No statistical adjustment was done for confounders	Lab test report was used to ascertain exposure	Yes*	There was no dropout during the study	Fair
Bhimwal *et* *al* 2017	Clinical examination, HAQ (Health Assessment Questionnaire) and DAS score used to define*	Patients were recruited from the dept. of medicine of MDM hospital. *	The 50 controls were enrolled from the same hospital*	The 50 controls were enrolled*	No statistical adjustment was done for confounders	Lab test report was used to ascertain exposure	Yes*	There was no dropout during the study	Fair
Jagtap *et* *al* 2017	ACR 2010 revised criteria was for definition*	Patients were recruited from the general OPD of Government Medical College, Nagpur*	The 26 controls were enrolled from the same hospital*	The 26 healthy participants were enrolled*	No statistical adjustment was done for confounders	Lab test report was used to ascertain exposure	Yes*	There was no dropout during the study	Fair
Latief *et* *al* 2017	Cases diagnosed as per revised criteria of ACR*	Patients who attended our hospital located in suburban region in India were*	The 25 controls were enrolled from the same hospital*	The 25 controls were enrolled*	No statistical adjustment was done for confounders	Lab test report was used to ascertain exposure	Yes*	There was no dropout during the study	Fair
Mateen *et* *al* 2017	Patients were diagnosed as per European League Against Rheumatism (EULAR) 2010*	Patients who visited the orthopaedics OPD of Jawaharlal Nehru Medical College, Aligarh*	50 controls were enrolled from the same hospital. *	The 50 controls were enrolled*	No statistical adjustment was done for confounders	Lab test report was used to ascertain exposure	Yes*	There was no dropout during the study	Fair
Meena *et* *al* 2018	Patients were diagnosed as per ACR-EULAR 2010 criteria*	Controls were enrolled from the department of Medicine of a tertiary care teaching hospital of Punjab, India*	50 controls were enrolled from the same hospital. *	The 50 controls were enrolled*	No statistical adjustment was done for confounders	Lab test report was used to ascertain exposure	Yes*	There was no dropout during the study	Fair
Rai *et* *al* 2017	Patients were diagnosed as per standard criteria*	Controls were enrolled from the department of orthopaedics and General medicine	40 controls were enrolled from the same hospital. *	The 40 controls were enrolled*	No statistical adjustment was done for confounders	Lab test report was used to ascertain exposure	Yes*	There was no dropout during the study	Fair
Sharma *et* *al* 2014	Patients were diagnosed as per ACR criteria*	Controls were enrolled from the same department as cases	20 controls were enrolled from the same hospital. *	The 20 controls were enrolled*	No statistical adjustment was done for confounders	Lab test report was used to ascertain exposure	Yes*	There was no dropout during the study	Fair

Good quality: 3 or 4 stars (F) in selection domain AND 1 or 2 stars in comparability domain AND 2 or 3 stars in outcome domain; Fair quality: 2 stars in selection domain AND 1 or 2 stars in comparability domain AND 2 or 3 stars in outcome/exposure domain; Poor quality: 0 or 1 star in selection domain OR 0 stars in comparability domain OR 0 or 1 stars in outcome/exposure domain.

### Observational studies

All the 12 observational studies were of fair quality since none of the studies used any statistical methods to adjust the potential confounders

### Randomised controlled trial

There were three randomised controlled trials among the eligible studies. All three studies had a high risk of bias for performance and detection bias. One study had a high risk of bias for selection bias since concealment allocation was not done. All three studies had a low risk of bias for selective reporting, incomplete outcome data and other biases. [Suppl. fig. 2]

### Outcomes

#### Serum 25(OH)D level in RA patients

**Figure 1:**
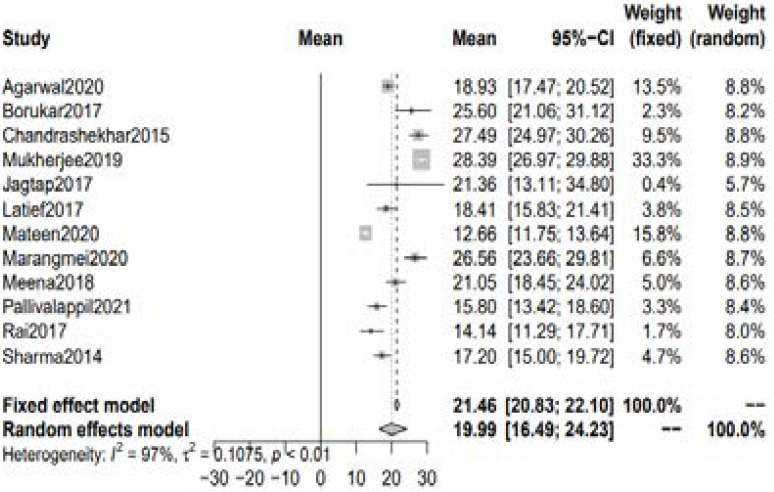
Mean serum Vitamin D levels in RA patients

A total of 11 studies with 678 patients of RA reported the serum level of vitamin D. The blood levels were taken before the initiation of any treatment in all these studies. The pooled estimate of mean serum 25(OH)D level in rheumatoid arthritis patients was 21.46 ng/ml [95% CI 20.83 to 22.10] using fixed effect model whereas 19.99 ng/ml [95% CI 16.49; 24.43] was seen with the random-effect model. [Fig. 1]

#### Prevalence of 25(OH)D level derangement in RA patients

**Figure 2:**
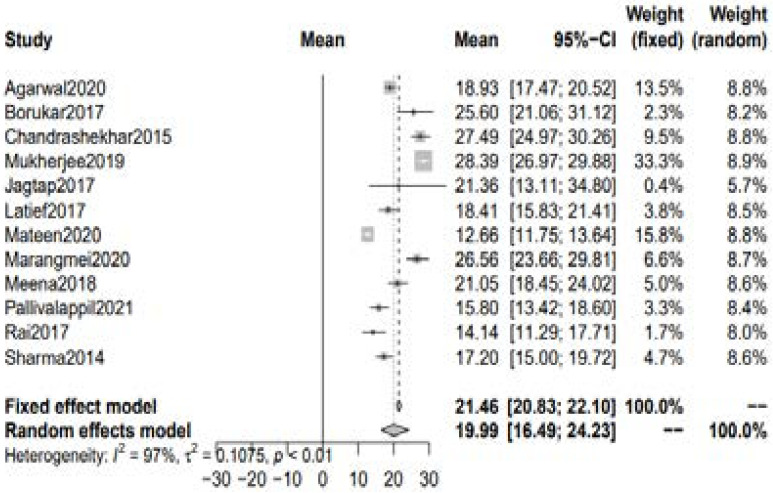
Incidence of low Vitamin D levels in RA patients

**Figure 3:**
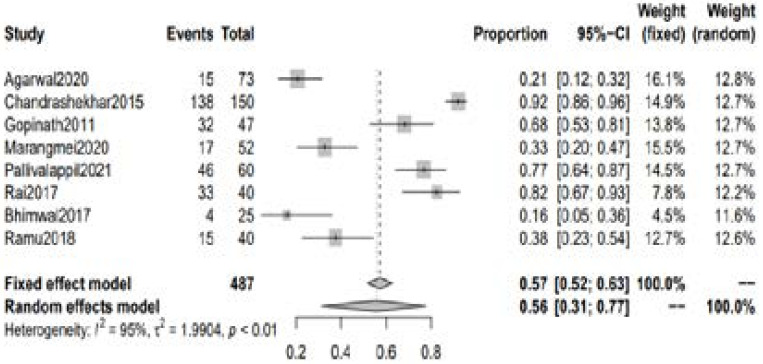
Incidence of Vitamin D deficiency in RA patients

**Figure 4:**
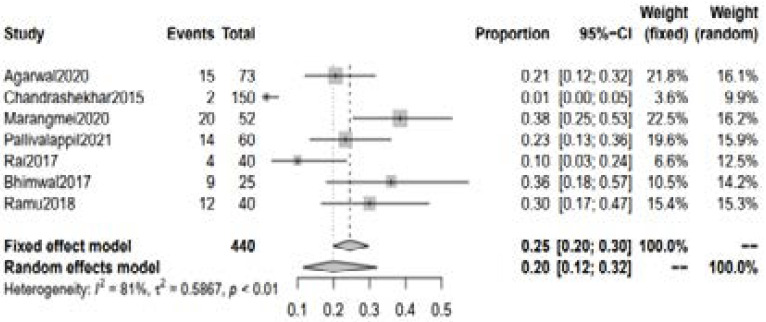
Incidence of Vitamin D insufficiency in RA patients

A total of 10 studies with 576 patients with RA reported abnormalities in 25(OH)D levels among the patients with RA. The pooled incidence of low Vitamin D (<30ng/dl) was 0.73 [95% CI 0.69 to 0.78] and 0.80 [95% CI 0.65 to 0.90] using fixed and random effect model respectively. [Fig. 2] The pooled incidence of vitamin D deficiency (<20ng/dl) was 0.57 [95% CI 0.52 to 0.63] using fixed effect model whereas 0.56 [95% CI 0.31 to 0.77] with random-effect model. [Fig. 3] Moreover, the pooled incidence of vitamin D insufficiency (20-30 ng/dl) was 0.25 [95% CI 0.20 to 0.30] with fixed effect model and 0.20 [95% CI 0.12 to 0.32] with random-effect model. [Fig. 4] Association between DAS score and Serum 25(OH)D level.

We found a significant difference (p<0.0001) in the serum 25(OH)D level among the patients with different DAS scores [ mild (<3.1), moderate (3.1 to 5.2) and severe (>5.2) using analysis of variance [ANOVA] test.

## Discussion

Our study highlights the incidence of deficiency and insufficiency of serum vitamin D in rheumatoid arthritis patients. Furthermore, we noted that the DAS score was significantly associated with serum 25(OH)D level which indicates that lower serum 25(OH)D level was observed in patients with higher DAS scores. This suggests that supplementing Vitamin D might help in improving the bone quality in rheumatoid arthritis. Vitamin D is not approved for the management of RA but is commonly used off label as a supplement to reverse bone quality due to the use of steroids in RA patients. In addition to its use as a nutritional supplement, Vitamin D has immunomodulatory activity and its deficiency is associated with most autoimmune disorders including rheumatoid arthritis [RA]. 3 RA is an inflammatory disease characterized by flares and remissions; flares being characterized by pain. Vitamin D metabolites have immunosuppressive effects and are known to maintain Th1 and Th2 balance to suppress the autoimmune response mediated by T cells, by regulating CD4+T cells production and activity and to overcome the effects of autoreactive T cells, Vitamin D increases the regulatory T cells' activity.^35^ Additionally, Vitamin D is found to downregulate the estrogen synthetase activity, hence controlling the autoimmune response and its deficiency may increase the risk for the development of RA. 36, 37

A study by Hiraki et al explored the role of Vitamin D insufficiency in the pathogenesis of RA. 38 Several RCTs were conducted to identify the efficacy of Vitamin D supplementation in RA. The outcomes were inconclusive, with most of the studies demonstrating lower serum 25(OH)D levels among patients with higher DAS scores whereas few others did not find any association between serum 25(OH)D levels and DAS scores.^37^, ^38^

In the present analysis, we found an association between 25(OH)D levels and DAS scores. A similar association was noted in a study from Raczkiewicz et al. They found a negative correlation between 25(OH)D level and DAS score but a positive correlation with the quality of life.^39^ Another meta-analysis by Lin j et al also noted a similar association between RA disease activity and serum 25(OH)D levels 8 whereas the meta-analyses by Song et al observed that higher consumption of Vitamin D led to a lower incidence of RA. 37

The wide prevalence of Vitamin D deficiency is seen in various studies carried in healthy as well as diseased populations despite India being a tropical country with adequate sunlight. 11,40 The included studies for the present analysis showed low serum 25(OH)D levels in rheumatoid arthritis patients compared to the healthy population.

Vitamin D is known to induce immunologic tolerance, consequently, its deficiency may alarm immune tolerance and induce the development of autoimmune diseases like RA. 41 Accordingly, vitamin D supplementation has been proposed to induce immune tolerance and, hence prevent the development of autoimmune diseases like RA. Nonetheless, RA patients are prone to develop osteoporosis and suffer from pain during a disease flare-up. 42, 43 Hence, vitamin D supplementation may be needed for the prevention of osteoporosis and pain relief in patients with RA.

Serum 25(OH)D behaves as a negative acute phase reactant with levels dropping during inflammatory reactions. In light of the current evidence, biochemical 25(OH) D measurements performed during the acute-phase response should be interpreted with caution. 44 Further research could help to clarify whether Vitamin D deficiency might be the cause or the consequence of chronic inflammatory diseases like RA.

Our study had several limitations that should be taken into account. First, only a few studies reported the DAS score which might lead to the overestimation of study results. The included studies enrolled a small number of participants, so the present association might change with a larger sample size. Secondly, we have not looked for the assay or methodology used to estimate 25(OH)D levels and the laboratory quality assurance as these parameters were not mentioned in the included studies. Furthermore, information regarding intake of Vitamin D supplements were also not mentioned which can confound the result outcomes. Despite these limitations, our study was the first study to report the incidence of Vitamin D deficiency in Indian patients with RA and we noted that almost half of the RA patients had Vitamin D deficiency. We also noted that the 25(OH)D level was inversely associated with the DAS score. However, these findings can only be confirmed in large scale RCTs before rationalising the use of Vitamin D in rheumatoid arthritis.

## Conclusions

Our study results aid to associate the Vitamin D deficiency state in patients with RA in India and exhibit an inverse relationship with RA disease activity. However, further observational studies and randomized controlled trials are warranted to establish the benefits of Vitamin D and to incorporate it into the guidelines for the management of RA.

## Figures and Tables

**Table 2:** Quality assessment of Cohort studies using NEWCASTLE OTTAWA SCALE (NOS)

Study ID [Author]	Selection				Comparability	Outcome			Overall Quality
	Representativeness of the exposed cohort	Selection of the non- exposed cohort	Ascertainment of exposure	Demonstration that outcome of interest was not present at start of study	Comparability of cohorts on the basis of the design or analysis controlled for confounders	Assessment of outcome	Was follow- up long enough for outcomes to occur	Adequacy of follow-up of cohorts	
Agarwal et al 2020	All patients attending the IPD and OPD departments, fulfilled ACR criteria were enrolled*	All patients attending the IPD and OPD fulfilled ACR criteria *	Lab test *	Yes *	No statistical adjustment was conducted for confounders	Based on lab report*	Yes, median follow was adequate in both the groups *	All patients were followed up. *	Fair
Marangmei et al 2010	RA patients attending Medicine OPD, Rheumatology OPD or admitted in the General Medicine wards at RIMS, Imphal. *	RA patients attending OPD or admitted at RIMS, Imphal. *	Lab test *	Yes *	No statistical adjustment was conducted for confounders	Based on lab report*	Yes, median follow was adequate in both the groups *	All patients were followed up. *	Fair
Pallivalappil et al 2021	All diagnosed patients with RA as per ACREULAR criteria attending Rheumatology, Medicine OP or Medicine wards.	All diagnosed patients with RA attending OPD or wards. *	Lab test *	Yes *	No statistical adjustment was conducted for confounders	Based on lab report*	Yes, median follow was adequate in both the groups *	All patients were followed up. *	Fair
Ramu et al 2018	Patients with newly diagnosed RA who met the ACR/EULAR 2010 classification	Patients diagnosed RA as per ACR/EULAR 2010. *	Lab test *	Yes *	No statistical adjustment was conducted for confounders	Based on lab report*	Yes, median follow was adequate in both the groups *	All patients were followed up. *	Fair

Good quality: 3 or 4 stars (F) in selection domain AND 1 or 2 stars in comparability domain AND 2 or 3 stars in outcome domain; Fair quality: 2 stars in selection domain AND 1 or 2 stars in comparability domain AND 2 or 3 stars in outcome/exposure domain; Poor quality: 0 or 1 star in selection domain OR 0 stars in comparability domain OR 0 or 1 stars in outcome/exposure domain.
